# Effects of Three Assertion Training Sessions on Schoolchildren’s Assertiveness and Mental Health

**DOI:** 10.3390/bs16081370

**Published:** 2026-08-10

**Authors:** Yuka Shiota, Naomi Fukushima, Yuki Hasegawa, Chiharu Tsuji

**Affiliations:** 1 Japan Society for the Promotion of Science, Tokyo 102-0083, Japan; 2 Department of Psychiatry and Neurobiology, Graduate School of Medical Science, Kanazawa University, Kanazawa 920-8640, Japan; 3 United Graduate School of Child Development, Osaka University, Kanazawa University, Hamamatsu University School of Medicine, Chiba University, and University of Fukui, Kanazawa 920-8640, Japanctsuji@med.kanazawa-u.ac.jp (C.T.); 4 Research Center for Child Mental Development, Kanazawa University, 13-1 Takara-machi, Kanazawa 920-8640, Japan

**Keywords:** assertiveness training, schoolchildren, mental health, strength and difficulties questionnaire, total difficulties score

## Abstract

As a crucial period for self and identity development, early adolescence may give rise to emotional issues due to inadequate methods of self-expression. Therefore, the development of self-expression during early adolescence may play a significant role in interpersonal relationships. “Assertion” refers to the ability to express one’s thoughts and emotions appropriately while simultaneously respecting those of others; thus, assertiveness training (AT) focuses on appropriate expression while also respecting others. Previous studies have shown AT programs’ effects over 1 to 6 months, but the minimum number of effective sessions has not been determined. Thus, this single-group study without a control group examined pre–post changes in 102 5th-grade students following three 45 min AT sessions on self-expression, requesting favors, and politely declining requests. Changes in assertiveness and mental health were assessed using the TK-Style Communication Skills and the Strength and Difficulties Questionnaire (SDQ). Results of available case analyses (depending on the outcome) showed a significant increase in assertiveness scores and decreases in both nonassertiveness scores and SDQ total difficulties scores. Overall, three short AT sessions were associated with favorable pre–post changes in assertiveness and mental health-related outcomes; however, the absence of a control group limits the causal interpretation.

## 1. Introduction

As a transition period between childhood and adulthood, adolescence is a critical developmental stage characterized by significant changes in identity formation, social relationships, and psychosocial well-being (Becht et al., 2016; Klimstra et al., 2010, 2016; Pfeifer & Berkman, 2018). During this period, individuals increasingly seek autonomy while becoming more sensitive to peer appraisal and social interactions (Meeus et al., 2005; Pfeifer et al., 2009). Simultaneously, emotional and behavioral difficulties such as irritability, depression, anxiety, and aggression often emerge or intensify (Carter et al., 2004).

Spanning the transition from late elementary school to middle school, early adolescence is particularly associated with increases in aggressive behavior and peer victimization (Williford et al., 2011). Although these difficulties are influenced by multiple developmental, family, school, and contextual factors, one potential contributing factor is that children may not have developed appropriate skills for self-expression and social communication. Difficulties in expressing one’s thoughts and needs effectively may contribute to emotional problems, including anxiety, depressive mood, and irritability. Previous research has shown that limited interpersonal skills are associated with increased stress and loneliness, ultimately affecting mental health negatively (Grant et al., 2007; Tse & Bond, 2004; Segrin, 2019; Starr et al., 2014).

Defined as the ability to express one’s thoughts and feelings appropriately while respecting others, assertiveness is a key component of adaptive interpersonal functioning (Fensterheim, 1972; Wilson & Gallois, 1993; Wolpe, 1990). Low levels of assertiveness have been associated with social anxiety, fear, and internalized distress (Noble & McGrath, 2005), whereas higher assertiveness levels have been linked to lower stress and depressive symptoms, as well as better self-esteem and interpersonal relationships (Shimizu et al., 2004; Sarkova et al., 2013). These findings suggest that assertiveness may function as a protective factor against emotional difficulties. Importantly, early adolescence can be a critical window for developing assertive communication skills (Khawar & Malik, 2016; Wang et al., 2016; Avşar & Ayaz Alkaya, 2017).

Assertiveness training (AT) is usually a series of training sessions designed to provide a basic understanding of assertiveness and help people acquire it through experiential learning. An AT program is a structured intervention technique to boost social relationships’ effectiveness and to express one’s thoughts and ideas appropriately (Turner et al., 2008; Gundogdu, 2012; Schroeder et al., 2012; Tavakoli et al., 2014). Effective training usually takes 1 to 6 months with numerous (8–12) sessions. Each session presents a different theme, structured with lectures, case studies, role-play, and discussions, or some combination. For example, a session may begin with an explanation of assertiveness and its importance in daily life, followed by examples of nonassertive (i.e., passive), aggressive, and assertive communication styles based on the session’s theme, such as “the ability to say no.” Assertive communication involves clearly expressing one’s needs while respecting others’ feelings, representing a healthy middle ground between passive and aggressive responses (Jandhyala & Kumar, 2024). Next, participants might engage in assertive communication role-play, aided by case studies of familiar situations. Because achieving assertiveness involves sincere, direct communication with others, it generally requires time and energy (Abdelaziz et al., 2020); however, incorporating practical training methods such as role-playing has yielded significant results (Pahmiah & Hasanati, 2024).

AT primarily aims to improve assertive communication skills through experiential learning. Improvements in assertiveness may facilitate more adaptive interpersonal communication and social relationships, which may contribute to psychological well-being. Previous studies have reported that AT is associated with improvements in self-esteem and self-efficacy (Boket et al., 2016; Yosep et al., 2024) and reductions in anxiety, stress, and depression (Eslami et al., 2016; Yosep et al., 2024). In addition, improved assertive communication has been associated with more adaptive interpersonal relationships (Boket et al., 2016) and prevents peer bullying (Avşar & Ayaz Alkaya, 2017). This may enable individuals to express their thoughts and defend their rights while respecting others’ rights (Eslami et al., 2016; Avşar & Ayaz Alkaya, 2017). Accordingly, AT may improve students’ relationships and lead to a more fulfilling school life.

Many studies have demonstrated AT’s effectiveness for school-aged children and adolescents (Watanabe & Ebata, 2015a; Watanabe & Ebata, 2015b; Eslami et al., 2016; Avşar & Ayaz Alkaya, 2017; Kaneko & Watanabe, 2019). However, these ATs lasted 4 to 12 weeks, with sessions once or twice a week. It may be difficult to implement such relatively long interventions during regular school schedules due to time constraints. Although short school-based interventions may be more feasible in practice, it remains unclear whether a short-duration AT program can produce measurable changes in assertiveness and emotional-behavioral outcomes. Clarifying whether several interventions produce meaningful change is therefore important for practical implementation in educational settings. To address this gap, the primary objective of the present study was to examine pre–post changes in assertiveness after three AT sessions among schoolchildren (5th grade, ages 10–11). In addition, pre–post changes in communication-related factors and mental health-related outcomes were examined.

## 2. Materials and Methods

### 2.1. Participants

Fifth-grade schoolchildren were recruited from one elementary school in Kanazawa City, Japan. In Japan, 99% of 5th graders are 10–11 years old. (https://www.moj.go.jp/isa/content/930004443.pdf (accessed on 15 November 2024)). The study was conducted in a moderately sized public elementary school. The fifth-grade cohort consisted of two classes, each with 34–35 students.

The researcher informed elementary school administrators and classroom teachers about the study’s purpose, content, questionnaires, and the training’s expected contribution. Classroom time was allotted for the study’s explanation and distribution of informed consent to students, to be signed at home by students and parents and handed back to classroom teachers within 10 days. No personal information other than attendance number and gender was obtained. All the school’s 140 (69 males; 71 females) 5th-graders were invited to participate in the study.

The study was approved by the Kanazawa University Medical Ethics Committee and conducted in accordance with local legislation and institutional requirements (2023-081-114344-2). Written informed consent was obtained from both the children and their parents for participation in the research.

### 2.2. Inclusion Criteria for Analysis

The AT program and pre-post tests were conducted as part of the regular curriculum for all fifth-grade students in the school. The only children who met the following criteria were included in the analyses: (1) agreed to use test scores for the analyses, (2) had parental consent, and (3) completed the pre- and post-tests and participated in all AT sessions. This study used a single-group pre–post design, without a control group. A control group was not included because of practical constraints in the school setting. In consultation with the collaborating school, both fifth-grade classes were required to complete the intervention and pre–post assessments on the same schedule, as implementing different intervention timings across classes would have interfered with the school’s annual instructional plan and classroom organization. Therefore, it was not feasible to establish a parallel comparison group. Power analysis was performed to determine the sample size: at least 34 participants [alpha (α) = 0.05, power (1 − β) = 0.80) (G*Power 3.1)] (Faul et al., 2007).

### 2.3. AT

To our knowledge, no widely standardized AT program has been designed specifically for children in Japan. However, there are some reports on AT programs, for instance, a sample lesson plan from Saitama Prefectural Education Center (2010). Based on their program, we restructured our three-session AT program as follows: (1) how to express your opinion, (2) how to request a favor, and (3) how to decline requests politely. With a classroom teacher’s support, the same researcher (1st author) facilitated each AT session, while the two other researchers (2nd and 3rd authors) performed role-play. AT was conducted from October to November 2023 and from June to July 2024. Students from two classes participated in each session.

### 2.4. Instruments

The TAKEN (TK) Style Communication Skills (for 5th and 6th grades) (Taken Publishing, Tokyo, Japan) was employed as a scale for measuring assertiveness (Watanabe, 2011). Japan has no standardized scale of assertiveness for school-aged children, and the scale above is the only standardized, commercially available psychological test. It consists of subscales to measure (1) self-expression, (2) factors related to self-expression, and (3) stress response. Subscale (1) demonstrates each child’s type of self-expression through 20 situations; children choose the self-expression that best applies to them, with the options categorized as assertive, nonassertive (i.e., passive), and aggressive. Each selected choice obtains one point, and scores are calculated for each category and summed. Higher scores for each communication style indicate a greater tendency to use that particular communication style in everyday situations. Subscale (2) measures factors related to self-expression, such as interpersonal anxiety, irrational beliefs, and understanding of basic human rights. All factors comprise seven items, and children choose the degree of their feelings and conditions on a 4-point scale (strongly agree = 3 points; somewhat agree = 2 points; somewhat disagree = 1 point; strongly disagree = 0 points). Scale (3) measures stress response with eight items, and children are evaluated on a 4-point scale (strongly agree = 3 points; somewhat agree = 2 points; somewhat disagree = 1 point; strongly disagree = 0 points). The TK-Style Communication Skills scale has demonstrated acceptable internal consistency (Cronbach’s α from 0.71 to 0.87) and construct validity (Watanabe, 2011). For scoring the scale, two types of self-assessment scoring sheets are used: Scoring Sheet A (Scale Score Tally Sheet) tallies the seven scale scores calculated during the workshop. Scoring Sheet B (Stage Conversion Sheet) converts the seven scale scores tallied in Scoring Sheet A into stages. A sample of the TK-Style Communication Skills scale is provided in Supplemental Materials.

Mental health was assessed using the Strength and Difficulties Questionnaire (SDQ) (Goodman, 1997), which consists of 25 items divided into five subscales: (1) Emotional Symptoms, (2) Conduct Problems, (3) Hyperactivity/Inattention, (4) Peer Relationship Problems, and (5) Prosocial Behavior. Each item is scored on a 3-point scale: 0 = not true; 1 = somewhat true; and 2 = certainly true. Each subscale’s scores are summed to give a score out of ten possible. The total difficulties score (TDS) is calculated by summing the first four subscales’ scores (Emotional Symptoms, Conduct Problems, Hyperactivity/Inattention, Peer Relationship Problems), giving a possible 40, with higher scores indicating greater difficulties. In contrast, higher scores on the Prosocial Behavior subscale indicate greater strengths. The Japanese version of the SDQ has been shown to have a stable five-factor structure with adequate reliability and validity (Moriwaki & Kamio, 2014). The child self-report version was used in the present study.

### 2.5. Procedure

**Figure 1:**
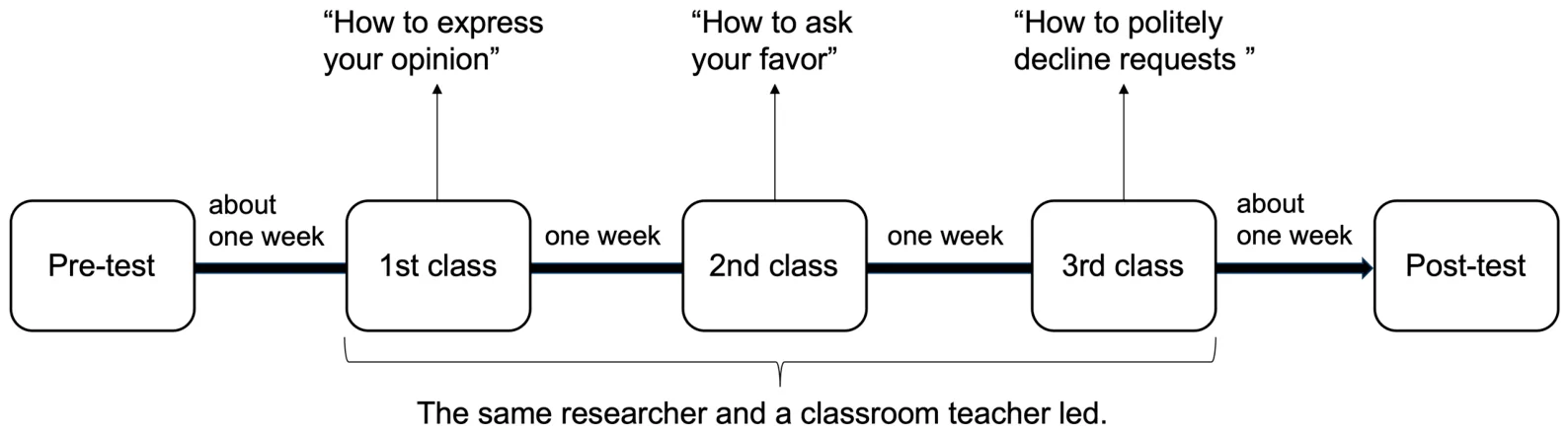
Time Flow of the Three-part Assertiveness Training for Schoolchildren.

**Figure 2:**
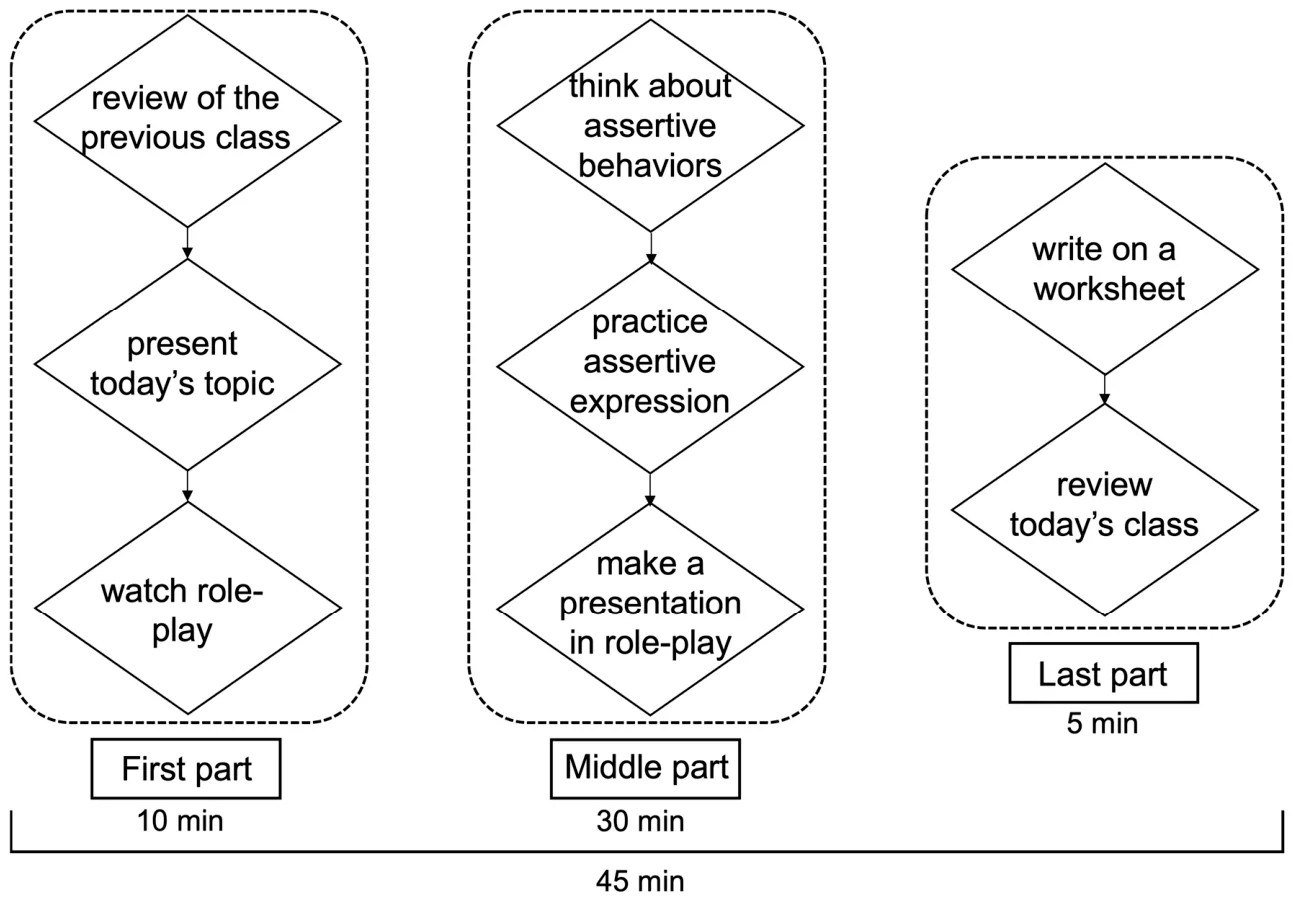
Time Flow of Assertiveness Training Sessions for Schoolchildren.

After consultation with the collaborating school, the intervention was designed as a three-session program. This number of sessions was considered feasible within the target grade’s annual schedule, available class activities, and integrated study periods, with each 45 min session corresponding to one standard class period in Japanese elementary schools. Approximately one week before AT began, the TK-Style Communication Skills workbook and SDQ were administered to the children as a child self-report in the classroom setting. The three-part training was given at 1-week intervals. Figure 1 shows the study’s flow. At the session’s beginning, an introduction to AT or a review of the previous session was provided. Then, a classroom teacher briefly introduced the session topic, while the researchers conducted role-play demonstrations and facilitated the learning activities. Classroom teachers primarily assisted with board writing, calling on students, and participating in role-play demonstrations when appropriate. To introduce the three self-expressions—assertive, nonassertive, and aggressive—the researchers wore masks of famous Japanese cartoon characters. The question “Which would you prefer for the next class recreation, dodgeball or fruit basket?” was asked, and the researchers then role-played two answer versions: nonassertive and aggressive. Harshly and aggressively, one researcher replied, “Fruit basket isn’t any fun!” The other researcher timidly and nonassertively replied, “… Well, we just played fruit basket the other day….” Next, the students discussed nonassertive and aggressive behaviors. Mid-session, a classroom teacher and researchers asked the students to think about assertive behaviors in relation to a topic aligned with the children’s actual situations. Students thought about assertive expressions in each situation and role-played the situations in pairs or groups. Subsequently, a few students shared their ideas of assertive expression by role-playing for the entire class. Nearing the session’s end, researchers asked students to complete a reflection worksheet by describing what they had learned during the session and how they would apply assertive communication in similar everyday situations (see Supplementary Materials for an example worksheet). A classroom teacher then reviewed the day’s session and announced the next. Each session lasted 45 min. Figure 2 depicts the sessions’ flow. To promote consistency in implementation, all sessions were led by the same primary facilitator (the first author), accompanied by one of the two co-authors, using standardized lesson plans, role-play scenarios, and identical worksheets. Each session followed the same standardized structure and was conducted in regular classroom settings with teacher support.

### 2.6. Data Analyses

All statistical analyses were conducted using Stata (ver. MP 16, Stata Corp., College Station, TX, USA). Pre–post changes were examined using paired *t*-tests for approximately normally distributed variables and Wilcoxon signed-rank tests for non-normally distributed variables. Between-group comparisons were performed using independent samples *t*-tests or Mann–Whitney U tests as appropriate.

For parametric analyses, mean differences with 95% confidence intervals (CIs) were reported, whereas for nonparametric analyses, medians and interquartile ranges (IQRs) were presented. Effect sizes were calculated for all outcomes: Cohen’s d for paired comparisons and r (Z/√N) for nonparametric tests.

Bonferroni correction was applied to adjust for multiple comparisons and reduce the risk of Type I errors, with adjusted significance thresholds set at *p* < 0.007 for TK-Style outcomes and *p* < 0.008 for SDQ outcomes. Raw *p*-values and statistical significance below corrected thresholds are reported in this study. A two-tailed significance level of *p* < 0.05 was used unless otherwise specified.

To assess potential attrition bias, participants with complete baseline data were compared with those with missing data using independent samples *t*-tests. Moreover, logistic regression analysis was conducted to examine whether baseline variables predicted participant exclusion. For the main analyses, an available-case approach was used, meaning that each outcome was analyzed using all participants with complete pre- and post-data for that specific variable. This approach assumes that missingness is not meaningfully related to the outcomes after accounting for the observed baseline data.

As additional analyses, mixed-effects models were used to examine potential differences in pre–post changes by gender, year, and class. To minimize the influence of missing data, these analyses were conducted using complete cases. The models included time (pre/post), subgroup variables (gender, year, or class), and their interactions as fixed effects, with random intercepts for participants. Accordingly, the primary analyses consisted of prespecified pre–post comparisons. In contrast, the mixed-effects models, subgroup analyses, and analyses examining alternative explanations for the observed changes were considered exploratory.

## 3. Results

**Figure 3:**
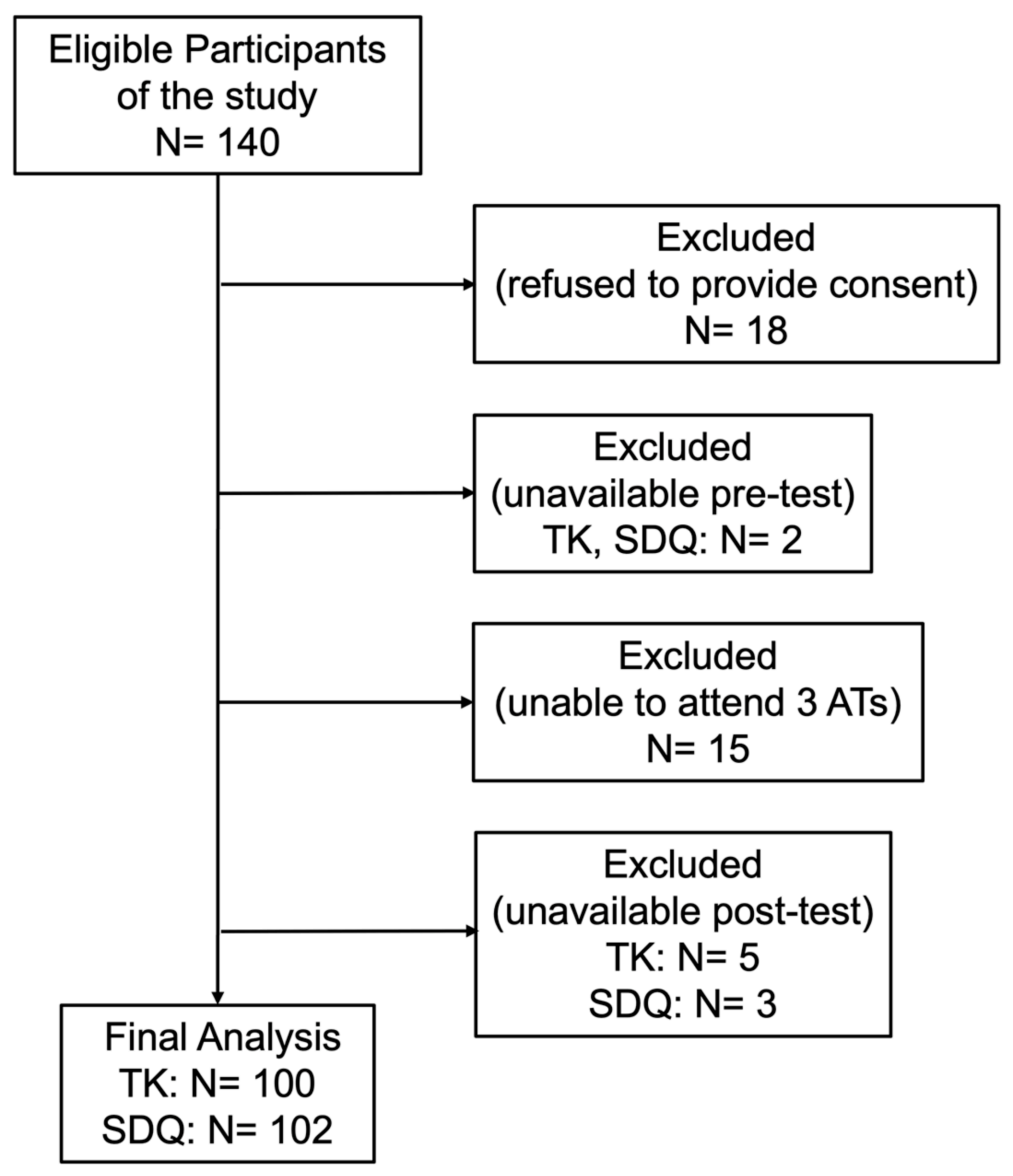
Flow diagram of participants.

Of the 140 eligible fifth-grade students, 102 individuals met the inclusion criteria for the final analyses. Although the final analytic samples comprised 100 participants for the TK-Style questionnaire and 102 participants for the SDQ, the sample size varied across the outcome variables because of item-level missing responses. Figure 3 presents a flow diagram of participant inclusion and exclusion criteria. No significant interaction effects were observed across years for either the TK-Style communication measures or the SDQ outcomes (all *p*s > 0.33). For the TK-Style measures, small main effects of year were observed for stress (*p* = 0.011) and interpersonal anxiety (*p* = 0.018). For SDQ outcomes, a year effect was detected for emotional symptoms (*p* = 0.046). Moreover, a significant class effect was observed for prosocial behavior in the SDQ (*p* = 0.002). However, these limited effects did not alter the findings’ overall pattern.

Missing data were more frequent in the TK-Style measures (available paired data ranged from 91 to 96 participants); SDQ measures showed greater data completeness (93–100 participants). This pattern was due to skipped items or partially completed questionnaires, particularly because the TK-Style scoring required complete responses for each scale. For each outcome, analyses were conducted using all participants with complete pre–post data; therefore, the sample sizes varied across variables.

To assess potential attrition bias, participants with complete data were compared with those with missing data at baseline (see Supplementary Table S1). No significant differences were observed for any variables. Additionally, logistic regression analysis showed that baseline variables did not significantly predict excluded participants (all *p* > 0.05; see Supplementary Table S2). These findings suggest that missing data were not systematically associated with the baseline characteristics. Accordingly, the assumption underlying the available-case analyses was considered reasonably plausible, although bias due to unmeasured factors cannot be ruled out.

**Figure 4:**
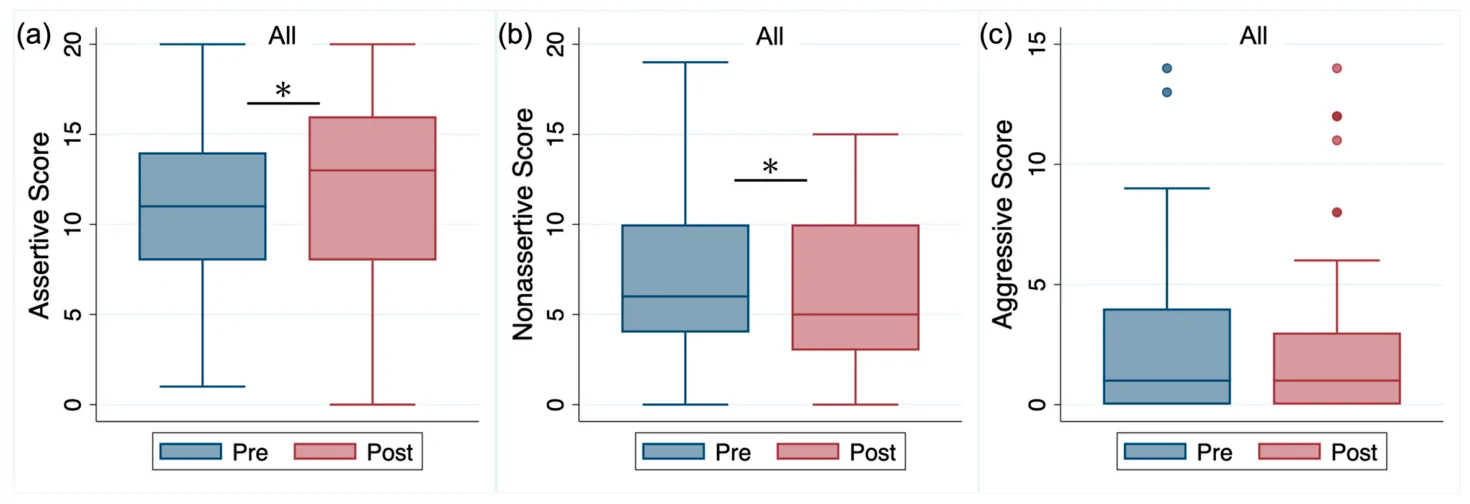
Boxplot of Variance Distribution in Assertiveness Scores. Note: (**a**) The boxplot of all children demonstrates a statistically significant difference in assertive scores between pre- and posttests. (**b**) The boxplot of all children indicates a statistically significant difference in nonassertive scores between pre- and posttests. (**c**) The boxplot of all children demonstrates no significant difference in aggressive scores between pre- and posttests. * indicates a statistically significant difference (*p* < 0.05).

Table 1 summarizes the changes in TK-Style outcomes. Significant improvements were observed in communication styles. Assertiveness scores increased, and nonassertiveness scores decreased significantly, with small-to-moderate effect sizes (Table 1; Figure 4a,b). These effects remained significant after the Bonferroni correction. In contrast, aggressive scores (Figure 4c), stress, and basic human rights showed no significant changes. Interpersonal anxiety and irrational beliefs showed nominal decreases, but these were not significant after the Bonferroni correction.

Mixed-effects analyses were conducted to examine whether intervention effects differed by gender, year, or class. These analyses were performed using complete cases with no missing data across TK-Style and SDQ variables (N = 71). Time (pre/post), group variables (gender, year, or class), and their interactions were included as fixed effects, with random intercepts for participants. No significant time × group interactions were observed for any TK-Style outcomes. Although a marginal interaction was observed between time and gender for nonassertive behavior (*p* = 0.054), it did not reach statistical significance. No significant interactions were observed for year (*p* = 0.162) or class (*p* = 0.889). Group-stratified results are presented in Supplementary Table S3.

For the SDQ scale, total difficulties scores (TDS) decreased significantly with a moderate effect size (Table 2). Among the subscales, emotional symptoms and hyperactivity/inattention showed significant reductions, which remained significant after Bonferroni correction. Although the median change in hyperactivity/inattention was 0, the Wilcoxon signed-rank test evaluates the distribution of paired differences rather than changes in the median alone; therefore, a statistically significant result may be observed even when the median remains the same. In contrast, conduct problems, peer relationship problems, and prosocial behavior showed no significant changes.

Additionally, mixed-effects analyses were conducted for SDQ outcomes using complete cases. No significant time × group interactions (gender or class) were observed for total difficulties or any subscales. Similarly, no significant time × year interactions were observed for most outcomes. However, a significant time × year interaction was observed for conduct problems (χ^2^ = 5.59, *p* = 0.018). Because this effect was isolated and not consistently observed across other outcomes, it should be interpreted cautiously. Group-stratified results are presented in Supplementary Table S4.

Additional analyses were conducted to examine potential alternative explanations for observed changes. The baseline scores were significantly associated with change scores for all primary outcomes (all *p* < 0.01), suggesting potential regression to the mean. No significant differences in change scores were observed between study years (2023 vs. 2024; all *p* > 0.05). Moreover, mixed-effects analyses revealed no significant differences in effects across gender, year, or class. These findings suggest that the observed pre–post changes were generally consistent across cohorts and classroom-level factors, although alternative explanations cannot be ruled out.

## 4. Discussion

This study examined the effects of three short AT sessions on 5th graders’ assertiveness and mental health. Following the intervention, assertiveness scores increased, and nonassertiveness scores decreased, along with the SDQ total difficulties scores. Although the effect sizes were generally small to moderate (*d* = 0.41 for assertiveness scores; *d* = 0.40 for nonassertiveness scores), these changes may still be meaningful in everyday classroom functioning (Cohen, 1988). Our results align with previous research indicating that in educational settings, assertiveness training is efficacious for increasing students’ assertiveness levels (Watanabe & Ebata, 2015a, 2015b; Eslami et al., 2016; Avşar & Ayaz Alkaya, 2017; Kaneko & Watanabe, 2019). Furthermore, whereas previous studies evaluated long-term AT programs (8–12 sessions), this study found favorable pre–post increases in assertiveness following three AT sessions. These findings suggest that a shorter AT program may be associated with favorable pre–post changes in assertiveness and mental health-related outcomes in Japanese schoolchildren.

Regarding the intervention procedures, the program also incorporated the masks of cartoon characters during role-play activities to create a playful, psychologically safe environment for practicing assertive communication. Although this approach may have increased engagement and reduced anxiety, this element’s specific contribution to the observed outcomes could not be determined. Future research may examine whether such engagement strategies differentially influence participation or learning among students with varying characteristics.

To the best of our knowledge, few studies have examined the mental health–related outcomes of AT using the SDQ. Following the intervention, total difficulties scores, emotional symptoms, and hyperactivity/inattention showed significant reductions. Our findings align with a previous study assessing children’s assertiveness and emotional functioning using the Child Emotional Adjustment Scale–Preschool Edition (CEAS-P) and SDQ, which reported associations between assertiveness and SDQ outcomes (Thorlacius & Gudmundsson, 2019). The reductions in total difficulties and hyperactivity/inattention were modest. These score changes may suggest potential relevance for school settings, but their practical significance remains uncertain. Given the modest effect sizes, reliance on child self-report, and immediate post-intervention assessment, these findings should be interpreted cautiously.

Although mixed-effects analyses indicated no significant gender differences in intervention effects, some descriptive patterns were observed. In particular, females showed significantly higher baseline nonassertive scores than males (t(99) = −3.07, *p* = 0.003), suggesting possible baseline differences in communication style. Moreover, both males and females demonstrated increases in assertiveness scores following the intervention, whereas reductions in nonassertiveness scores appeared to be more pronounced in females. However, these patterns should be interpreted cautiously because the time × gender interaction for nonassertive behavior did not reach statistical significance (χ^2^ = 3.72, *p* = 0.054). Thus, these findings are better interpreted as reflecting baseline tendencies rather than evidence of differential intervention effects by gender. The subgroup analyses by gender, year, and class should be considered exploratory. Because the sample size became smaller after stratification, small subgroup differences may have been difficult to detect. Therefore, the absence of significant subgroup interactions should not be interpreted as definitive evidence that the intervention effects were equivalent across all subgroups. Nevertheless, no clear differences in pre–post changes were observed across gender, year, or class. Although these exploratory findings require confirmation in larger multi-school studies, the consistency of effects across gender cohorts and classes suggests that the intervention may be implemented across different classroom contexts without requiring substantial adaptation based on gender composition or class-specific characteristics. This may facilitate broader integration into routine classroom practice, as educators could apply the same core intervention approach across classes while making only minor contextual adjustments where necessary.

Contrary to previous studies (Watanabe & Ebata, 2015a, 2015b; Eslami et al., 2016), we did not observe improvement in stress and interpersonal anxiety. Notably, in Eslami et al. (2016), compared to the control group, the experimental group had lower stress scores 2 months after AT than immediately after AT. In Watanabe and Ebata (2015a, 2015b), no significant difference was found in stress between pre- and posttests, but the 3-month follow-up revealed a significant reduction. Additionally, interpersonal anxiety scores were significantly lower in the pre-follow-up comparison than in the pre–post comparison (Watanabe & Ebata, 2015a, 2015b). These results suggest that psychological effects to lower stress and anxiety may be achieved long-term. Possible explanations for the lack of significant changes in variables related to stress and anxiety may be as follows. First, this study involved a short intervention of three sessions, and it is believed that assertiveness changes occur in the order of skill learning, behavioral adjustment, and psychological state (Tenison & Anderson, 2016; Boutcher & Rotella, 1987). Accordingly, changes in anxiety and stress are expected to manifest in time rather than immediately. Second, because improvements in anxiety and stress may emerge as secondary effects of enhanced interpersonal communication, longer follow-up may be needed to capture these changes. Previous studies have examined the psychological effects of stress and anxiety 2 to 3 months after AT completion, suggesting that delayed effects should be considered.

Notably, our participants’ mean pretest scores in TK-Style Communication Skills were higher than the mean pretest scores of children in a previous study (Watanabe & Ebata, 2015a). Children who originally had high pretest assertive scores may be effective with short-term AT sessions, whereas children who had low pretest assertive scores may need more AT sessions. Further research is necessary to examine the relationship between the pretest assertiveness score and the effective number and duration of AT sessions. To examine potential alternative explanations for outcomes that showed significant pre–post changes, several additional analyses were conducted. Mixed-effects models indicated that baseline scores significantly predicted change scores for assertiveness, nonassertiveness, and total difficulties, suggesting that regression to the mean may have contributed to observed changes. This possibility is particularly important given the single-group pre–post design, in which participants with more extreme baseline scores may naturally move toward the average at follow-up. Additional analyses showed no clear differences in pre–post changes across gender, year, or class. However, these analyses do not rule out the regression to the mean or other non-intervention-related explanations. Therefore, the observed changes should be interpreted cautiously.

Other alternative explanations should also be considered. Because the same self-report measures were administered before and after the intervention, testing effects may have influenced the responses by increasing familiarity with the questionnaire items. Furthermore, demand characteristics or social desirability may have affected responses, as students may have inferred the intended goals of the training and responded in ways they perceived as favorable to the teachers or researchers. Classroom-level influences, such as peer dynamics and teacher expectations, and seasonal variation may also shape students’ responses. Furthermore, because post-testing was conducted shortly after the final session, the observed changes may partly reflect short-term response shifts or temporary increases in awareness rather than sustained behavioral changes. The observed improvements may also reflect changes in students’ self-perception or understanding of assertive communication rather than actual behavioral changes in everyday school settings.

Beyond assertiveness training, the present findings may be interpreted within the broader context of school-based social-emotional learning (SEL). SEL programs can promote social competence, emotional regulation, positive classroom behavior, and academic adjustment (Durlak et al., 2011; Taylor et al., 2017). Although the present intervention focused specifically on assertive communication rather than a comprehensive SEL curriculum, the observed pre–post changes were broadly consistent with the educational goal of strengthening students’ interpersonal communication skills within regular classroom activities. These findings suggest that brief communication-focused interventions, including assertiveness training, may be a practical component of broader school-based socioemotional support.

Educationally, although the observed effect sizes were generally small to moderate, even modest improvements in assertive communication skills may have practical implications for students’ everyday school experiences, as these skills are used repeatedly in classroom interactions. Being able to express opinions, communicate discomfort, and politely decline requests may help students navigate peer interactions more effectively and reduce interpersonal stress. Assertiveness may also facilitate help-seeking behavior with teachers, such as asking questions or requesting support when experiencing academic or social difficulties. Thus, even modest improvements in assertive communication may contribute to improved peer relationships, greater classroom participation, and overall school adjustment.

From a practical perspective, teachers and school counselors may incorporate brief assertiveness-focused activities, such as role-play and communication exercises, into existing classroom activities. Because the present intervention was implemented within regular class periods without substantial additional burden, it may represent a feasible approach for schools with limited time and resources. At a broader level, these preliminary findings may inform educational policymakers when considering the potential role of brief AT in school-based socioemotional support programs. Nevertheless, broader implementation should await confirmation from controlled studies with longer follow-up and behavioral validation.

This study has several limitations. First, the single-group pre–post design limits causal interpretation. Future studies using randomized, waitlist-controlled, delayed-intervention, or other controlled designs are needed to confirm intervention effects.

Second, the relatively small sample size and recruitment from a single school and grade in Kanazawa City may limit the generalizability of the findings, as contextual factors (e.g., school culture, regional characteristics, classroom environment, and teacher or student differences) may have influenced both the implementation of the intervention and the observed outcomes. In addition, because the study was conducted within a single Japanese cultural and educational context, the findings may not be generalizable to settings where norms surrounding assertive communication differ.

Third, although missing data were examined and no clear baseline differences were identified, the use of available-case analyses may still have introduced bias if missingness was related to unmeasured factors. This may have affected the precision of the estimated pre–post changes. Future studies should consider methods such as multiple imputation or full-information approaches to better address the missing data.

Fourth, behavioral changes following the intervention were not directly assessed, and the outcomes were based on child self-report questionnaires. Although the SDQ is a well-validated screening instrument for emotional and behavioral difficulties, it is not a direct measure of change in clinical mental health. Accordingly, the observed reductions in SDQ scores should be interpreted as changes in self-reported emotional and behavioral difficulties rather than as definitive improvements in mental health. More broadly, the observed improvements may reflect changes in children’s self-perception or understanding of assertive communication rather than confirmed behavioral changes. Future studies should consider incorporating teacher-rated outcomes, parent reports, peer nominations, direct behavioral observations, and longer-term follow-up assessments to determine whether improvements in self-reported assertiveness are reflected in sustained behavioral changes.

Fifth, intervention fidelity was only partially assessed. Although several procedures were used to standardize implementation across classrooms, including a single primary facilitator, standardized lesson plans, and identical teaching materials, no formal fidelity checklist or quantitative adherence rating was employed. Overall, subtle variations in implementation across classrooms cannot be completely ruled out, which may have influenced the consistency of the intervention delivery.

Finally, although some improvements were observed, the extent to which these effects generalize across grade levels and are maintained over time remains unclear. Future studies involving larger, multi-school samples and longitudinal designs are needed to confirm the consistency and generalizability of the findings and to validate the persistence and practical application of assertiveness skills.

## 5. Conclusions

This study provides preliminary evidence that a short-term, in-class AT program may be associated with favorable pre–post changes in early adolescents’ self-expression and mental health-related outcomes. Additionally, although descriptive gender-related patterns were observed, no significant gender differences were found in the pre–post changes. Specifically, three AT sessions were associated with increased assertiveness scores and reduced self-reported difficulties, suggesting potential feasibility within the time constraints of school settings. The findings highlight the value of school-based interventions that can be integrated into regular classroom activities without imposing substantial additional demands, thereby offering teachers and school counselors a practical approach to supporting children’s socioemotional development. Nevertheless, the long-term effects remain unclear and should be examined in future studies. Future research using randomized controlled trials, longer follow-up periods, and larger multi-school samples across different age groups is needed to better establish the long-term impact and generalizability of this intervention.

## Figures and Tables

**Table 1:** Pre–post Changes in TK-Style Communication Skills Outcomes.

Variables	N	Pre	Post	Change (95% CI)/Median [IQR]	Test	*p*	Sig. (Adjusted)	Effect Size
Assertive	91	10.75 (4.23)	12.41 (4.64)	+1.66 [0.81, 2.51]	Paired t	<0.001	Yes	d = 0.41
Nonassertive	91	7.20 (4.13)	5.80 (4.11)	−1.40 [−2.12, −0.67]	Paired t	<0.001	Yes	d = 0.40
Aggressive	91	1 [0–4]	1 [0–3]	0 [−1 to 0]	Wilcoxon	0.138	No	r = 0.16
Stress	93	10.56 (6.57)	9.74 (7.23)	−0.82 [−1.76, 0.13]	Paired t	0.089	No	d = 0.18
Interpersonal Anxiety	91	11.31 (6.12)	10.36 (6.31)	−0.95 [−1.69, −0.20]	Paired t	0.013	No	d = 0.27
Irrational Belief	94	11.41 (3.99)	10.57 (4.16)	−0.84 [−1.56, −0.12]	Paired t	0.022	No	d = 0.24
Basic Human Rights	96	26.65 (4.64)	26.72 (4.51)	+0.07 [−0.66, 0.81]	Paired t	0.845	No	d = 0.02

Note: Values are presented as means (standard deviations [SDs]) for normally distributed variables and medians (IQR) for non-normally distributed variables. For parametric analyses, mean differences with 95% confidence intervals (CIs) are reported; for nonparametric analyses, median changes with interquartile ranges (IQRs) are reported. Paired *t*-tests were used for normally distributed variables, and Wilcoxon signed-rank tests for non-normally distributed variables. Effect sizes were calculated as Cohen’s d for paired *t*-tests and r (Z/√N) for Wilcoxon signed-rank tests. Bonferroni correction was applied (*p* < 0.007 for TK-Style outcomes). Raw *p*-values are reported, and statistical significance after correction is indicated in the table.

**Table 2:** Pre–post Changes in SDQ Outcomes.

Variable	N	Pre	Post	Change (95% CI)/Median [IQR]	Test	*p*	Sig. (Adjusted)	Effect Size
TDS	93	12.90 (5.22)	11.41 (5.18)	−1.49 [−2.12, −0.87]	Paired t	<0.001	Yes	d = 0.49
Emotional Symptoms	99	4.03 (2.34)	3.34 (2.44)	−0.69 [−1.07, −0.30]	Paired t	0.001	Yes	d = 0.36
Conduct Problems	99	2 [1–3]	2 [1–3]	0 [−1 to 0]	Wilcoxon	0.285	No	r = 0.11
Hyperactivity/Inattention	100	4 [3–5]	3 [2–5]	0 [−1 to 0]	Wilcoxon	0.0004	Yes	r = 0.35
Peer Relationship Problems	99	2.10 (1.67)	2.05 (1.71)	−0.05 [−0.35, 0.25]	Paired t	0.738	No	d = 0.03
Prosocial Behavior	100	6.19 (1.78)	6.11 (1.93)	−0.08 [−0.37, 0.21]	Paired t	0.592	No	d = 0.05

Note: Values are presented as mean (SD) for normally distributed variables and median (IQR) for non-normally distributed variables. For parametric analyses, mean differences with 95% confidence intervals (CIs) are reported; for nonparametric analyses, median changes with interquartile ranges (IQRs) are reported. Paired *t*-tests were used for normally distributed variables, and Wilcoxon signed-rank tests were used for non-normally distributed variables. Effect sizes were calculated as Cohen’s d for paired *t*-tests and r (Z/√N) for Wilcoxon signed-rank tests. Bonferroni correction was applied (*p* < 0.008 for SDQ outcomes). Raw *p*-values are reported, and statistical significance after correction is indicated in the table.

## Data Availability

The original contributions presented in this study are included in the article/Supplementary Materials. Further inquiries can be directed to the corresponding author.

## Supplementary Materials

The following supporting information can be downloaded at: https://www.mdpi.com/article/10.3390/bs16081370/s1, Table S1. Comparison of Baseline Characteristics Between Participants With and Without Complete Paired Data. Table S2. Logistic Regression Analysis Predicting Excluded Participants. Table S3. Pre–post Changes in TK-Style Communication Skills Outcomes by Gender. Table S4. Pre–post Changes in SDQ Outcomes by Gender.

## References

[B1-behavsci-16-01370] Abdelaziz E. M., Diab I. A., Ouda M. M. A., Elsharkawy N. B., Abdelkader F. A. (2020). The effectiveness of assertiveness training program on psychological wellbeing and work engagement among novice psychiatric nurses. Nursing Forum.

[B2-behavsci-16-01370] Avşar F., Ayaz Alkaya S. (2017). The effectiveness of assertiveness training for school-aged children on bullying and assertiveness level. Journal of Pediatric Nursing.

[B3-behavsci-16-01370] Becht A. I., Nelemans S. A., Branje S. J., Vollebergh W. A., Koot H. M., Denissen J. J., Meeus W. H. (2016). The quest for identity in adolescence: Heterogeneity in daily identity formation and psychosocial adjustment across 5 years. Developmental Psychology.

[B4-behavsci-16-01370] Boket E., Bahrami M., Kolyaie L., Hosseini S. (2016). The effect of assertiveness skills training on reduction of verbal victimization of high school students. International Journal of Humanities and Cultural Studies.

[B5-behavsci-16-01370] Boutcher S. H., Rotella R. J. (1987). A psychological skills educational program for closed-skill performance enhancement. The Sport Psychologist.

[B6-behavsci-16-01370] Carter A. O., Saadi H. F., Reed R. L., Dunn E. V. (2004). Assessment of obesity, lifestyle, and reproductive health needs of female citizens of Al Ain, United Arab Emirates. Journal of Health, Population and Nutrition.

[B7-behavsci-16-01370] Cohen J. (1988). Statistical power analysis for the behavioral sciences.

[B8-behavsci-16-01370] Durlak J. A., Weissberg R. P., Dymnicki A. B., Taylor R. D., Schellinger K. B. (2011). The impact of enhancing students’ social and emotional learning: A meta-analysis of school-based universal interventions. Child Development.

[B9-behavsci-16-01370] Eslami A. A., Rabiei L., Afzali S. M., Hamidizadeh S., Masoudi R. (2016). The effectiveness of assertiveness training on the levels of stress, anxiety, and depression of high school students. Iranian Red Crescent Medical Journal.

[B10-behavsci-16-01370] Faul F., Erdfelder E., Lang A. G., Buchner A. (2007). G*Power 3: A flexible statistical power analysis program for the social, behavioral, and biomedical sciences. Behavior Research Methods.

[B11-behavsci-16-01370] Fensterheim H. (1972). The initial interview. Clinical behavior therapy.

[B12-behavsci-16-01370] Goodman R. (1997). The strength and difficulties questionnaire: A research note. Journal of Child Psychology and Psychiatry.

[B13-behavsci-16-01370] Grant D. M., Gayle Beck J., Farrow S. M., Davila J. (2007). Do interpersonal features of social anxiety influence the development of depressive symptoms?. Cognition and Emotion.

[B14-behavsci-16-01370] Gundogdu R. (2012). Effect of the creative drama-based assertiveness program on the assertiveness skill of psychological counsellor candidates. Educational Sciences: Theory and Practice.

[B15-behavsci-16-01370] Jandhyala S., Kumar N. (2024). Everyday assertiveness and its significance for overall mental well-being. Universal Journal of Public Health.

[B16-behavsci-16-01370] Kaneko S., Watanabe R. (2019). A practical study on classroom assertion training for children who need support for communication: Through the creation of lessons utilizing work that enhances TK style communication skills—Communication ni shien ga hitsuyo na jido heno gakkyu tani no assertion training ni kansuru jissen kenkyu—TK shiki communication ryoku wo takameru work wo katsuyo shita jyugyo dukuri wo toshite. Ibaraki Daigaku Kyoiku Gakubu Kiyo 68.

[B17-behavsci-16-01370] Khawar R., Malik F. (2016). Bullying behavior of Pakistani pre-adolescents: Findings based on Olweus questionnaire. Pakistan Journal of Psychological Research.

[B18-behavsci-16-01370] Klimstra T. A., Hale W. W. I., Raaijmakers Q. A., Branje S. J., Meeus W. H. (2010). Identity formation in adolescence: Change or stability?. Journal of Youth and Adolescence.

[B19-behavsci-16-01370] Klimstra T. A., Kuppens P., Luyckx K., Branje S., Hale W. W., Oosterwegel A., Koot H. M., Meeus W. H. J. (2016). Daily dynamics of adolescent mood and identity. Journal of Adolescent Research.

[B20-behavsci-16-01370] Meeus W., Iedema J., Maassen G., Engels R. (2005). Separation–individuation revisited: On the interplay of parent–adolescent relations, identity and emotional adjustment in adolescence. Journal of Adolescence.

[B21-behavsci-16-01370] Moriwaki A., Kamio Y. (2014). Normative data and psychometric properties of the strengths and difficulties questionnaire among Japanese school-aged children. Child and Adolescent Psychiatry and Mental Health.

[B22-behavsci-16-01370] Noble T., McGrath H. (2005). Emotional growth--helping children and families “bounce back”. Australian Family Physician.

[B23-behavsci-16-01370] Pahmiah P., Hasanati N. (2024). Intervention to improve assertive behavior in the educational world: A literature review. Journal of Scientific Research, Education, and Technology (JSRET).

[B24-behavsci-16-01370] Pfeifer J. H., Berkman E. T. (2018). The development of self and identity in adolescence: Neural evidence and implications for a value-based choice perspective on motivated behavior. Child Development Perspectives.

[B25-behavsci-16-01370] Pfeifer J. H., Masten C. L., Borofsky L. A., Dapretto M., Fuligni A. J., Lieberman M. D. (2009). Neural correlates of direct and reflected self-appraisals in adolescents and adults: When social perspective-taking informs self-perception. Child Development.

[B26-behavsci-16-01370] Saitama Prefectural Education Center (2010). Yori yoi ningen kankei wo hagukumu assertion training shido program no kaihatsu ni kansuru chosa kenkyu. In Heisei 22 Nen do Sogo Kyoiku center Kenkyu Hokoku Sho, 344.

[B27-behavsci-16-01370] Sarkova M., Bacikova-Sleskova M., Orosova O., Madarasova Geckova A., Katreniakova Z., Klein D., van den Heuvel W., van Dijk J. P. (2013). Associations between assertiveness, psychological well-being, and self-esteem in adolescents. Journal of Applied Social Psychology.

[B28-behavsci-16-01370] Schroeder B. A., Messina A., Schroeder D., Good K., Barto S., Saylor J., Masiello M. (2012). The implementation of a statewide bullying prevention program: Preliminary findings from the field and the importance of coalitions. Health Promotion Practice.

[B29-behavsci-16-01370] Segrin C. (2019). Indirect effects of social skills on health through stress and loneliness. Health Communication.

[B30-behavsci-16-01370] Shimizu T., Kubota S., Mishima N., Nagata S. (2004). Relationship between self-esteem and assertiveness training among Japanese hospital nurses. Journal of Occupational Health.

[B31-behavsci-16-01370] Starr L. R., Hammen C., Connolly N. P., Brennan P. A. (2014). Does relational dysfunction mediate the association between anxiety disorders and later depression? Testing an interpersonal model of comorbidity. Depression and Anxiety.

[B32-behavsci-16-01370] Tavakoli P., Setoodeh G., Dashtbozorgi B., Komili-Sani H., Pakseresht S. (2014). The influence of assertiveness training on self-esteem in female students of government high schools of Shiraz, Iran: A randomized controlled trial. Nursing Practice Today.

[B33-behavsci-16-01370] Taylor R. D., Oberle E., Durlak J. A., Weissberg R. P. (2017). Promoting positive youth development through school-based social and emotional learning interventions: A meta-analysis of follow-up effects. Child Development.

[B34-behavsci-16-01370] Tenison C., Anderson J. R. (2016). Modeling the distinct phases of skill acquisition. Journal of Experimental Psychology: Learning, Memory, and Cognition.

[B35-behavsci-16-01370] Thorlacius Ö., Gudmundsson E. (2019). The development of the children’s emotional adjustment scale–preschool version. Journal of Psychoeducational Assessment.

[B36-behavsci-16-01370] Tse W. S., Bond A. J. (2004). The impact of depression on social skills: A review. Journal of Nervous & Mental Disease.

[B37-behavsci-16-01370] Turner N. E., Macdonald J., Somerset M. (2008). Life skills, mathematical reasoning and critical thinking: A curriculum for the prevention of problem gambling. Journal of Gambling Studies.

[B38-behavsci-16-01370] Wang W., Brittain H., McDougall P., Vaillancourt T. (2016). Bullying and school transition: Context or development?. Child Abuse & Neglect.

[B39-behavsci-16-01370] Watanabe R. (2011). Zaidan hojin tanaka kyoiku kenkyujyo hen TK shiki communication ryoku wo takameru work.

[B40-behavsci-16-01370] Watanabe R., Ebata A. (2015a). A practical study on improvement of the communicative competence of children (1)—A trial of the assertion training in the elementary school—Jido no communication noryoku wo takameru tame no jissen kenkyu. Ibaraki Daigaku Kyoiku Gakubu Kiyo.

[B41-behavsci-16-01370] Watanabe R., Ebata A. (2015b). A practical study on improvement of the communicative competence of children (2)—A trial of the assertion training in the elementary school—jido no communication noryoku wo takameru tame no jissen kenkyu. Ibaraki Daigaku Kyoiku Gakubu Kiyo.

[B42-behavsci-16-01370] Williford A. P., Brisson D., Bender K. A., Jenson J. M., Forrest-Bank S. (2011). Patterns of aggressive behavior and peer victimization from childhood to early adolescence: A latent class analysis. Journal of Youth and Adolescence.

[B43-behavsci-16-01370] Wilson K., Gallois C. (1993). Assertion and its social context.

[B44-behavsci-16-01370] Wolpe J. (1990). The practice of behavior therapy.

[B45-behavsci-16-01370] Yosep I., Suryani S., Mediani H. S., Mardhiyah A., Maulana I., Hernawaty T., Hazmi H. (2024). A scoping review of assertiveness therapy for reducing bullying behavior and its impacts among adolescents. Journal of Multidisciplinary Healthcare.

