# Supplementary material for: Effects of Three Assertion Training Sessions on Schoolchildren’s Assertiveness and Mental Health
Source: Behav Sci (Basel). 2026 Aug 10;16(8):1370. doi: 10.3390/bs16081370 (PMC13509681; doi:10.3390/bs16081370)
Supplement: Supplementary file 1 [file behavsci-16-01370-s001.zip › behavsci-4361603-supplementary.pdf]

Supplemental Materials

Supplementary 1

Example questions and answers for Scale (1)

Question 1

You have fought with a friend over something small. What do you do in this situation?

a. Your friend is wrong, so you complain to them or ignore them.

b. You reflect on your actions, apologize, and talk to your friend about it.

c. You remain quiet until your friend comes to apologize.

Question 2

You had plans to meet up with a friend, but they did not show up at the agreed time. When you got tired of waiting and were about to leave, your friend finally showed up. What do you do in this situation?

a. You do not mention the delay and pretend to not be bothered by it.

b. You ask the reason for the delay and then express how you feel about it.

c. Your friend is at fault for not keeping their word, so you ask for an apology.

Question 3

You score well on a test and are extremely happy. What do you do in this situation?

a. You suppress the desire to urge to share the news and then celebrate alone.

b. You compare scores with friends around you and show off.

c. You express honestly your happiness while being mindful of your friends' feelings.

Supplementary 2

A) **Example questions for Scale (3). Participants selected their answers from the 4-point scale.**

Example 1. For some reason, you sometimes feel that you are all alone.

Example 2. For some reason, you feel sad every day.

Example 3. You do not feel like doing anything.

B) **Example questions for Scale (2). Participants selected their answers from the 4-point scale.**

Example 1. You are concerned that your friends talk bad about you.

Example 2. You are conscious of blushing when people look at you.

Example 3. You try to avoid places where a lot of people gather.

C) **Example questions for Scale (2). Participants selected their answers from the 4-point scale.**

Example 1. When your friends ask you to do something, you must always accept.

Example 2. You are allowed to say “No” clearly when you do not want to do something.

Example 3. You are allowed to speak your mind even if your opinion differs from others’ opinions.

Supplementary 3

Expressing Your Thoughts and Feelings to Your Friends

Grade: ______ Class: ______ Student No.: ______

〇Let’s value both your own feelings and the feelings of your friends!

Assertive communication

Aggressive communication

「Fruit basket isn’t any fun!」

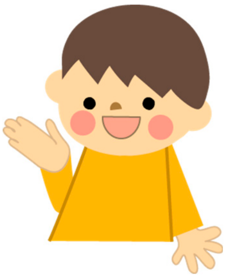

Nonassertive communication

「... Well, we just played fruit basket the other day....」

After today’s lesson, write what you learned, how you felt, or what you thought.

Supplementary Tables

Supplementary Table S1. Comparison of Baseline Characteristics Between Participants With and Without Complete Paired Data.

Variables	Included N	Included Mean (SD)	Excluded N	Excluded Mean (SD)	Mean difference [95% CI]	t-value	p-value
Assertive (pre)	91	10.75 (4.23)	7	8.14 (6.26)	−2.60 [−6.02, 0.81]	−1.51	0.134
Nonassertive (pre)	91	7.20 (4.13)	7	8.14 (6.62)	0.95 [−2.42, 4.31]	0.56	0.579
Aggressive (pre)	91	2.05 (2.66)	7	3.71 (3.55)	1.66 [−0.46, 3.78]	1.55	0.124
TDS (pre)	93	12.90 (5.22)	6	12.00 (6.03)	−0.90 [−5.30, 3.50]	−0.41	0.685

Note: Included participants were defined as those with complete paired data (pre–post) for each outcome variable, whereas excluded participants had missing paired data. Sample sizes change across variables due to missing data.

Supplementary Table S2. Logistic Regression Analysis Predicting Excluded Participants.

Predictor	Odds ratio [95% CI]	z	p-value
Assertive (pre)	0.79 [0.59, 1.05]	-1.63	0.10
Nonassertive (pre)	0.86 [0.66, 1.13]	-1.08	0.279
TDS (pre)	1.02 [0.87, 1.19]	0.23	0.814

Note: Odds ratios and 95% confidence intervals (CIs) are reported. No baseline variables significantly predicted excluded participants.

Supplementary Table S3. Pre–post Changes in TK-Style Communication Skills Outcomes by Gender.

Variables	Group	N	Pre	Post	Change (95% CI) / median [IQR]	Test	p	Sig.	Effect size
**Assertive**	Male	45	11.58 (4.10)	12.80 (4.58)	+1.22 [0.08, 2.37]	Paired t	0.037	No	d = 0.32
	Female	46	9.93 (4.25)	12.02 (4.73)	+2.09 [0.79, 3.38]	Paired t	0.002	Yes	d = 0.48
**Nonassertive**	Male	45	5.62 (3.26)	5.04 (4.02)	−0.58 [−1.49, 0.34]	Paired t	0.209	No	d = 0.19
	Female	46	8.74 (4.33)	6.54 (4.10)	−2.20 [−3.29, −1.10]	Paired t	0.0002	Yes	d = 0.59
**Aggressive**	Male	45	2 [0–4]	1 [0–3]	0 [−1 to 0]	Wilcoxon	0.023	No	r = 0.34
	Female	46	1 [0–2]	0 [0–2]	0 [−1 to 0]	Wilcoxon	0.867	No	r = 0.02
**Stress**	Male	43	10.14 (6.21)	8.63 (5.98)	−1.51 [−2.98, −0.05]	Paired t	0.043	No	d = 0.32
	Female	50	10.92 (6.90)	10.70 (8.09)	−0.22 [−1.47, 1.03]	Paired t	0.724	No	d = 0.05
**Interpersonal Anxiety**	Male	43	10.44 (5.93)	9.79 (5.87)	−0.65 [−1.75, 0.45]	Paired t	0.24	No	d = 0.18
	Female	48	12.08 (6.24)	10.88 (6.70)	−1.21 [−2.24, −0.18]	Paired t	0.023	No	d = 0.34
**Irrational Belief**	Male	44	11.91 (4.50)	11.11 (4.60)	−0.80 [−1.82, 0.23]	Paired t	0.125	No	d = 0.24
	Female	50	10.98 (3.48)	10.10 (3.72)	−0.88 [−1.92, 0.16]	Paired t	0.095	No	d = 0.24
**Basic Human Rights**	Male	46	26.17 (5.40)	26.30 (4.99)	+0.13 [−0.95, 1.21]	Paired t	0.809	No	d = 0.04

	Female	50	27.08 (3.81)	27.10 (4.03)	+0.02 [−1.03, 1.07]	Paired t	0.97	No	d < 0.01

Note*:* Values are presented as means (standard deviations [SD]) for normally distributed variables and median (interquartile range [IQR]) for non-normally distributed variables.
Mean changes are shown with 95% confidence intervals for parametric tests, and median changes with IQRs for nonparametric tests.
Paired t-tests were used for normally distributed variables, and Wilcoxon signed-rank tests were used for non-normally distributed variables.
Cohen's d was calculated using the standard deviation of the difference scores for paired t-tests, and effect size r was calculated as Z/√N for Wilcoxon tests.
Bonferroni correction was applied, with an adjusted significance threshold of $p < .007$.
Raw p-values are reported, and statistical significance after correction is indicated in the table.

Supplementary Table S4. Pre–post Changes in SDQ Outcomes by Gender.

Variable	Group	N	Pre	Post	Change (95% CI) / median [IQR]	Test	p	Sig. (adjusted)	Effect size
TDS	Male	42	12.79 (4.30)	11.24 (4.22)	− 1.55 [− 2.52, − 0.58]	Paired t	0.003	Yes	d = 0.50
	Female	51	13.00 (5.91)	11.55 (5.94)	− 1.45 [− 2.30, − 0.60]	Paired t	0.001	Yes	d = 0.48
Emotional Symptoms	Male	45	3.58 (2.11)	3.00 (2.11)	− 0.58 [− 1.10, − 0.05]	Paired t	0.032	No	d = 0.33
	Female	54	4.11 (2.77)	3.33 (2.73)	− 0.78 [− 1.34, − 0.21]	Paired t	0.008	No	d = 0.38
Conduct Problems	Male	47	2 [1–3]	2 [1–3]	0 [− 1 to 0]	Wilcoxon	0.323	No	r = 0.14
	Female	52	2 [1–3]	2 [1–3]	0 [− 1 to 0]	Wilcoxon	0.614	No	r = 0.07
Hyperactivity/Inattention	Male	46	4 [3–5.5]	3 [2–5]	0 [− 1 to 0]	Wilcoxon	0.027	No	r = 0.33
	Female	54	4 [3–5]	3 [2–5]	0 [− 1 to 0]	Wilcoxon	0.006	Yes	r = 0.38

Peer Relationship Problems	Male	46	2.50 (1.75)	2.48 (1.46)	− 0.02 [− 0.45, 0.41]	Paired t	0.92	No	d = 0.01
	Female	53	2.45 (1.58)	2.38 (1.56)	− 0.08 [− 0.51, 0.36]	Paired t	0.727	No	d = 0.05
Prosocial Behavior	Male	47	6.13 (1.65)	6.13 (1.86)	0.00 [− 0.48, 0.48]	Paired t	1	No	d = 0.00
	Female	53	6.25 (1.91)	6.09 (2.00)	− 0.15 [− 0.52, 0.22]	Paired t	0.415	No	d = 0.11

Note: Values are presented as means (SD) for normally distributed variables and medians (IQR) for non-normally distributed variables.
Mean changes are shown with 95% CIs for parametric tests, and median changes with IQRs for nonparametric tests.
Paired t-tests were used for normally distributed variables, and Wilcoxon signed-rank tests were used for non-normally distributed variables.
Cohen's d was calculated using the SD of the difference scores for paired t-tests, and effect size *r* was calculated as $Z/\sqrt{N}$ for Wilcoxon tests.
Bonferroni correction was applied, with an adjusted significance threshold of $p < .008$.
Raw p-values are reported, and statistical significance after correction is indicated in the table.
